# X-ray reflecto-interferometer based on compound refractive lenses

**DOI:** 10.1107/S1600577519007896

**Published:** 2019-08-12

**Authors:** S. Lyatun, D. Zverev, P. Ershov, I. Lyatun, O. Konovalov, I. Snigireva, A. Snigirev

**Affiliations:** a Immanuel Kant Baltic Federal University, 14 Nevskogo, 236041 Kaliningrad, Russian Federation; b European Synchrotron Radiation Facility, 71 avenue des Martyrs, 38043 Grenoble, France

**Keywords:** spatial resolution, temporal resolution, interferograms, reflecto-interferometers, compound refractive lenses, X-ray interferometry

## Abstract

An X-ray amplitude-splitting interferometer based on compound refractive lenses, which operates in the reflection mode, is proposed and realized. The idea of a reflecto-interferometer is to use a very simplified experimental setup where a focused X-ray beam reflected from parallel flat surfaces creates an interference pattern in a wide angular range. The functional capabilities of the interferometer were experimentally tested at the European Synchrotron Radiation Facility (ESRF) ID06 beamline in the X-ray energy range from 10 keV to 15 keV. The main features of the proposed approach, high spatial and temporal resolution, were demonstrated experimentally. The reflections from free-standing Si_3_N_4_ membranes, gold and resist layers were studied. Experimentally recorded interferograms are in good agreement with our simulations. The main advantages and future possible applications of the reflecto-interferometer are discussed.

## Abstract 2

An X-ray amplitude-splitting interferometer based on compound refractive lenses, which operates in the reflection mode, is proposed and realized.

## 1. Introduction   

Interferometry is a well established set of methods in many fields of science and industry. Interferometers in visible-light optics are widely used for diagnostics and metrology because of the availability of lasers with a high degree of spatial coherence. Regarding the development of X-ray interferometry, until recently, the lack of coherence was compensated by the use of perfect crystal optics, where dynamical diffraction was employed. Because of the coherent interaction of X-rays with a three-dimensional crystal structure, the crystal becomes an intrinsically coherent device and therefore does not require spatial coherence of the radiation. A classic example of this type of interferometer is the Bonse–Hart interferometer, which utilizes both transmission and surface Bragg reflection components (Bonse & Hart, 1965 ▸; Hart & Bonse, 1970 ▸). The Bonse–Hart interferometer was used for the first time to record phase-contrast X-ray computed tomograms for biomedical and material science applications (Momose *et al.*, 1996 ▸). The X-ray standing-wave technique is an X-ray interferometric method combining diffraction with a multitude of spectroscopic techniques, which makes it extremely powerful for obtaining information about virtually all properties of surfaces and interfaces on the atomic scale (Batterman & Cole, 1964 ▸).

The appearance of highly coherent X-ray sources, such as third-generation synchrotrons and X-ray free-electron lasers, allowed researchers to realize classical schemes of wave-splitting interferometers. Because of its very small size, the coherence of the X-ray source was measured in several different ways, namely with double slits (Leitenberger *et al.*, 2001 ▸, 2004 ▸; Paterson *et al.*, 2001 ▸; Lyubomirskiy, Snigireva & Snigirev, 2016 ▸), double mirrors (Fezzaa *et al.*, 1997 ▸; Leitenberger & Pietsch, 2007 ▸; Lyubomirskiy *et al.*, 2015 ▸) and double prisms (Lang & Makepeace, 1999 ▸; Suzuki, 2004 ▸). The laser-like properties of synchrotron beams were used to develop paraxial schemes of X-ray interferometry, like grating interferometers, which were used for phase-contrast imaging of objects with a micrometre resolution (David *et al.*, 2002 ▸; Weitkamp *et al.*, 2005 ▸).

It should be noted that new X-ray sources, besides having a small source size, have the unique property of microradian angular divergence, which was successfully used for the implementation of compound refractive lenses (CRLs). CRLs were demonstrated, then reached maturity, and are now widely employed on virtually all synchrotrons in the energy range from 3 keV to 200 keV (Snigirev *et al.*, 1996 ▸; Lengeler, Schroer, Richwin *et al.*, 1999 ▸; Lengeler, Schroer, Tümmler *et al.*, 1999 ▸; Aristov *et al.*, 2000 ▸; Schroer *et al.*, 2005 ▸; Snigireva & Snigirev, 2006 ▸; Snigirev, Snigireva *et al.*, 2007 ▸; Snigirev & Snigireva, 2008 ▸; Terentyev *et al.*, 2015 ▸; Polikarpov *et al.*, 2015 ▸). In addition to traditional micro-focusing applications, refractive optics can provide various beam-conditioning functions: condensers, microradian collimators, low-band-pass filters (Vaughan *et al.*, 2011 ▸), high-harmonics rejecters (Polikarpov *et al.*, 2014 ▸) and beam-shaping elements (Zverev *et al.*, 2017 ▸). The field of applications of CRLs was extended to Fourier optics as well as coherent diffraction and imaging techniques (Drakopoulos *et al.*, 2005 ▸; Petukhov *et al.*, 2006 ▸; Bosak *et al.*, 2010 ▸; Ershov *et al.*, 2013 ▸; Byelov *et al.*, 2013 ▸; Simons *et al.*, 2015 ▸; Falch *et al.*, 2017 ▸). Moreover, CRLs allow transfer to the X-ray length scale for some approaches well developed in visible-light optics. The most relevant CRLs applicability is ‘in-line interferometry’: bi-lens and multi-lens interferometers based on Si planar refractive lenses, working on the principle of a wavefront-splitting interferometer, are successfully developing (Snigirev *et al.*, 2009 ▸, 2014 ▸; Lyubomirskiy, Snigireva, Kohn *et al.*, 2016 ▸).

In this article, we present an amplitude-splitting interferometer operating in the reflection mode employing CRLs. A coherent monochromatic X-ray beam, focused by a CRL, impinges on a thin film at a grazing angle. This converging fan of radiation incoming on a sample surface provides a range of grazing angles. For each grazing angle, the rays reflected from the front and rear boundaries of the film will interfere, and as a result the diffracted intensity in the range of exit angles equal to the angular range of the incident fan will generate an interference pattern representing fringes of equal inclination, which in the case of X-rays are called Kiessig fringes (Kiessig, 1931 ▸). This interference fringe pattern can be recorded in a single interferogram or a single shot using a high-resolution two-dimensional area detector.

Seemingly straightforward, our approach possesses two features, which will be explained in this article. First, the most structurally representative part of the interference pattern, which is in fact a specular X-ray reflectivity, can be simultaneously and quickly measured in a very simplified experimental setup without the need to rotate the specimen or detector. The second feature of the proposed configuration is spatial resolution, allowing one to probe different regions on the film with an accuracy in the order of several tens of micrometres. Moreover, the probing area can be quickly changed without modifying the experimental setup by moving the sample in the vertical direction.

There have been attempts to use a convergent monochromatic X-ray beam to measure reflectivity (Naudon *et al.*, 1989 ▸; Niggemeier *et al.*, 1997 ▸; Miyazaki *et al.*, 2000 ▸) but our approach is much simpler and more flexible because of the unique properties of CRLs. For example, it is possible to perform reflectivity measurements in a wide energy range without realignment of the optical scheme and also the ability to easily change the irradiation area of the sample (Vaughan *et al.*, 2011 ▸). In addition, both methods have been demonstrated using laboratory sources and are difficult to implement on synchrotron sources. X-ray reflectivity with a convergent polychromatic X-ray beam (Matsushita *et al.*, 2013 ▸) was realized on a synchrotron but the approach we proposed with a monochromatic beam solves the problem of changing the refractive index with the wavelength. Most recently, quick X-ray reflectivity (XRR) for thin-film growth was demonstrated using polycapillary optics to produce a converging beam (Joress *et al.*, 2018 ▸). However, this approach has poor spatial resolution, requirements to the sample are the same as in the classical XRR, and energy tunability is lacking. It should also be noted that an X-ray reflection interface microscope based on Fresnel zone plates was implemented for imaging of the spatial variation of the local X-ray reflectivity (Fenter *et al.*, 2006 ▸).

Therefore, the scope of this article is to provide a general concept of the proposed reflecto-interferometer, to report the experimental results obtained using this interferometer and, finally, to give trends to future applications that can be realized with an interferometer. The theory of X-ray reflectometry will be briefly considered in connection with our case.

## 2. Basics of X-ray reflecto-interferometry theory   

Within the framework of classical wave optics, electromagnetic plane waves, including visible wavelength, incident on an ideal flat surface will in general be partially reflected and partially transmitted as refracted waves. The relationship between the amplitudes of the incident, reflected and refracted (transmitted) waves is described by the Fresnel equations, while the direction of the refracted waves in the material with refractive index *n* is defined by Snell’s law (Born & Wolf, 1999 ▸; Parratt, 1954 ▸).

In the X-ray region, the complex refractive index *n* is slightly less than unity and can be expressed as *n* = 1 − δ + *i*β, where δ and β are the refractive index decrement and the absorption index, respectively. This entails that X-rays impinging on a flat surface can be totally reflected with a reflectivity *R_I_* = 1. The condition to observe the total external reflection is that the grazing angle θ (defined here as the angle between the incident ray and the surface) should be less than the critical angle θ_c_. Since δ is of the order of 10^−5^–10^−6^, which is much less than unity, the critical angle is extremely small (of the order of a few milliradians) and can be estimated from Snell’s law as θ_c_ ≃ (2δ)^1/2^, where θ_c_ is in radians. This assumption is valid for reflection between air with refractive index *n* = 1 and another material with refractive index *n* = 1 − δ, where absorption is neglected, *i.e.* β = 0. Above the critical angle θ_c_, the reflectivity *R_I_* decreases very rapidly in proportion to θ^−4^, which can be derived from the Fresnel equations using the small-angles approximation.

The classical X-ray reflectivity technique is based on measurements of the intensity of X-rays specularly reflected from a surface as a function of the grazing angle. Thin films on a surface can give rise to oscillations of the X-ray intensity with a variation of the grazing angle, providing information on the thickness, roughness and density of thin films. From optics of visible light, it is known that when a thin film of a constant thickness is illuminated with a convergent beam of monochromatic light the reflectivity curve can be obtained without scanning the sample incident angle. Likewise, if the incident X-ray beam is focused by compound refractive lens then the representative part of the reflectivity curve can be obtained in a single interferogram. This is possible because the converging fan of X-rays, which are incoming on the film, provides a sufficient range of grazing angles.

Let us consider a thin film of constant thickness *d* placed in the air and illuminated with a convergent beam of monochromatic light produced by a focusing CRL. For grazing angles of greater than the critical angle θ_c_, reflections occur both on the upper and lower surface of the film because of the penetration of X-rays inside the film. The contribution of multiple reflections inside the film can be neglected for angles greater than two critical angles. As a result, a thin film forms two divergent X-ray beams reflected from both surfaces, which interfere in the overlapping region. The outcome of the interference is intensity oscillations. The intensity distribution of the fringes in the interference pattern is angular dependent and is determined by the corresponding contributions of the reflected rays to interference at each point where the interference is observed.

Interference at any point is characterized by the phase difference of only two waves reflected from the upper and lower surfaces that come to this point. Taking into account the phase shift by π arising from reflection from an optically denser air medium on the bottom of the film, the value of the phase difference φ in the zeroth approximation is given by the formula
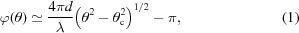
where θ > θ_c_ is the grazing angle of the ray reflected at the upper surface and λ is the X-ray wavelength. This formula is valid for reflection from a thin film satisfying the condition 2*d*/θ_c_ ≪ *L*, where *L* is the distance from the film to the observation point. The left side of the condition is nothing more than the maximum size of the longitudinal illumination of the film by the focused beam. In practice, interference is observed at distances of several tens of centimetres, while the size of the illuminated region is several tens of micrometres, so this condition is always satisfied.

Thus, the illumination of a thin film by a focused beam produces a reflectivity curve, fully described in the context of the classical approach, where a collimated incident beam is considered. Therefore, the constructive interference condition for φ = 2*m*π can be expressed as
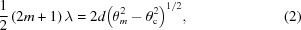
where *m* is a non-negative integer and θ_*m*_ is the angle at which the *m*th interference maximum is located. It should be noted that this distribution of fringes is described by the film thickness, which can be defined by consideration of two neighbouring interference maxima,
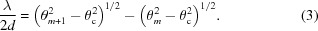
Employing this formula, the greatest angular distance Δθ = θ_*m*__+1_ − θ_*m*_ between two successive interference fringes at angles θ_*m*_ ≫ θ_c_ can be expressed as Δθ ≃ λ/2*d*. The derived formula allows estimating of the minimum and maximum film thickness, which can be measured by X-ray reflectometry techniques based on CRLs.

As already mentioned, the focused beam allows one to observe a part of the reflectivity curve without a scanning procedure. The range of grazing angles is given by the numerical aperture of the lens, which can be estimated as NA = *A*_eff_/*f*, where *A*_eff_ and *f* are the effective aperture and the focal distance of the lens, respectively. From this it follows that the minimum number of fringes observed in the reflecto-interferogram can be expressed as NA/Δθ. As for the minimum thickness of the film, which can be studied by our method, it is also determined by this ratio, but only when it is equal to unity. Considering that the typical numerical aperture of the lens is less than a few mrad, the proposed method is effective for investigating films with thicknesses of several tens of nanometres.

With respect to the maximum thickness of the film that can be analysed by our technique, the film thickness is limited by the instrumental angular resolution, which is defined as the ratio of the spatial resolution of the detector to the distance from the film to the detector. The instrumental angular resolution should be better than Δθ, which is usually achievable. For example, considering a detector with a spatial resolution of 1 µm located 1 m away from the film, it is possible to achieve a typical instrumental angular resolution of about 1 µrad, meaning that theoretically our technique allows the studying of films with thicknesses up to several tens of micrometres.

## 3. Experiment   

The experimental tests of the proposed reflecto-interferometer were carried out at the Micro Optics Test Bench at the European Synchrotron Radiation Facility (ESRF) ID06 beamline (Snigirev, Hustache *et al.*, 2007 ▸). The beam was produced by an in-vacuum undulator, and desired X-ray energies were selected by a cryogenically cooled Si(111) double-crystal monochromator with Δλ/λ ≃ 10^−4^. The effective source size, which was measured by the boron-fibre technique, was 40 µm and 900 µm in the vertical and horizontal direction, respectively (Kohn *et al.*, 2000 ▸).

**Figure 1:**
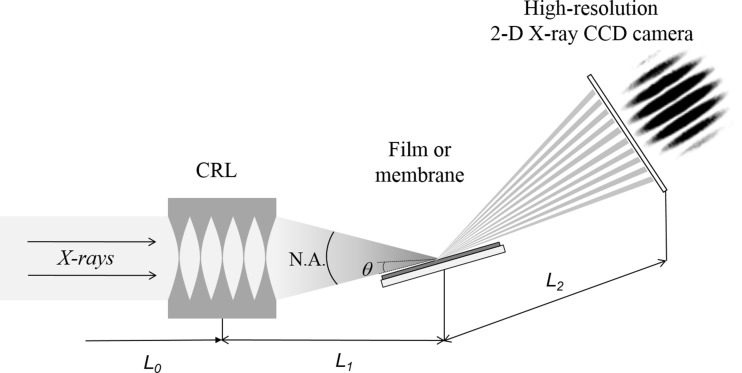
Schematic view of the proposed reflecto-interferometer.

Beryllium two-dimensional and linear CRLs were used to generate the converging X-ray beam. A CRL focuses the beam at a distance *L*_1_ = *f* *L*_0_/(*L*_0_ − *f*), where *L*_0_ is the source-to-lens distance. The lens focal distance is defined as *f* = *R*/2*Nδ*, where *N* is the number of elements in the lens, *R* is the curvature radius of the parabola apex and δ is the decrement of the refractive index. The CRL was located at *L*_0_ = 58 m from the source and was mounted on motorized stages with all the necessary translation and rotation movements. The reflecto-interferometer was tested at X-ray energies in the range 10–15 keV, so the number of individual lenses in the CRL and, correspondingly, the focal length of the lens varied. Therefore, the CRL parameters and lens imaging distances will be detailed in the sections describing the experimental results. The sample tilted at a certain angle θ with respect to the axis of the focused beam was mounted on a stage that was equipped with high-resolution angular and linear movements (Fig. 1 ▸). The sample stage was placed at the distance *L*_1_ from the CRL. The reflected intensity at the corresponding 2θ angle was recorded by a high-resolution two-dimensional charge-coupled device (CCD) camera with a spatial resolution of about 1.3 µm (0.645 µm pixel size), located at a distance *L*_2_ from the sample. The sample-to-detector distance was also adapted depending on the energy used.

The first step in obtaining the reflecto-interferogram is the alignment of the CRL, the precise definition of its focal length and the size of the focal spot. The CRL, mounted on motorized stages with all the necessary translation and rotation movements, was placed in the beam path and centred along the optical axis. Measurements of the focal spot were carried out using the CCD camera, which was placed at the calculated (expected) focal distance. Images of the focused beam were recorded and a focal spot was measured [full width at half-maximum (FWHM)] by scanning the CCD detector along the optical axis within the depth of the lens focus. Precise measurements of the dimensions of the focused beam were performed by the knife-edge technique (Suzaki & Tachibana, 1975 ▸). The distance at which the minimum focal spot was detected corresponds to the lens imaging distance *L*_1_. It was carefully measured and at this distance a sample was located.

The second step to obtaining the interference pattern is to align the sample. The membrane, which is placed almost horizontally on the goniometer, is raised vertically until the primary beam is intercepted by the sample, so that just a half beam is visible at the CCD detector. Then, starting from this initial angular position, the sample is tilted in a direction allowing one of the two edges of the sample to be observed on the detector. Next, the sample is tilted in the opposite direction to see the other edge on the detector. The average angular value between these angular positions corresponds to the condition of parallelism between the primary beam and the sample surface, and the total external reflection can be seen in the CCD camera as a bright strip located above or below the sample surface, depending on the experimental conditions. Usually, it is necessary to repeat these two steps several times to make sure that the sample surface is properly aligned and only then can the angle θ between the primary beam and the surface be considered as θ_0_ = 0. Such preparations allowed us to set the inclination angles of the membrane accurately during the reflectivity measurement.

For processing and visual demonstration of all of the experimental results presented below, numerical calculations were performed using the *LEPTOS7* program from Bruker (Bruker AXS GmbH, Karlsruhe, Germany) and software developed by us for modelling X-ray optics based on a wave-optics approach. Numerical simulations were performed with exactly the same parameters as in the experiment. It is worth noting that, for a better presentation, experimentally obtained and simulated reflecto-interferograms are represented on a logarithmic grey scale.

### 3.1. Test of Si_3_N_4_ membranes   

To verify the concept of the proposed reflecto-interferometer, silicon nitride membranes were used, which are commercially available from Silson Ltd, UK (‘standard silicon nitride membrane windows’ http://www.silson.com/index.html?content=standard). Membranes are typically fabricated by depositing an amorphous Si_3_N_4_ film on a bulk silicone substrate followed by etching down bevels into the silicon wafer, such that a small window consisting of the Si_3_N_4_ film remains. The size of the Si supporting frame of the membranes was 10 mm × 10 mm with a thickness of 200 µm. The thicknesses of the Si_3_N_4_ membranes that were used in our experiments were 200 nm, 500 nm and 1000 nm with the same membrane window 2.5 mm × 2.5 mm in size.

An X-ray beam of 14.4 keV energy was focused by a two-dimensional CRL comprising 71 individual beryllium lenses with a parabola apex radius *R* of 50 µm. The CRL imaging distance *L*_1_ at this energy was 23 cm, which was where the sample was placed. Interferograms were recorded with a high-resolution two-dimensional camera, which was located at a distance *L*_2_ of 42 cm behind the sample. The distance was selected so that the corresponding angular size of the entrance aperture of the camera was almost the same as the NA of the CRL, which was about 1.5 mrad. This allowed one to inscribe the entire reflected beam in the field of view of the camera and provided optimal angular resolution equal to 1.5 µrad. The critical angle for Si_3_N_4_ membranes at this energy was θ_c_ = 2.6 mrad, and, consequently, reflections from the upper and lower interfaces for inclination angles above θ_c_ will interfere resulting in the creation of interference fringes. Since relatively thick membranes have been studied, the numerical values for the period of the fringes are denoted in microradians. Interferograms were recorded at the different membrane inclination angles θ ranging from 1.7 mrad to 8.7 mrad. Because of the significant decrease of the reflectivity with the increase of the inclination angle, the time of obtaining the interference patterns varied from 0.05 s to 1 s.

The CRL produced a focal spot with dimensions of 0.2 µm × 3.2 µm in vertical and horizontal directions, respectively. Depending on the inclination angle, the beam projection on the sample surface along the beam propagation varied from 150 µm to 20 µm. For example, at a grazing angle of about 4.3 mrad, it is ∼50 µm. This means that the lens-based reflecto-interferometer provides a high spatial resolution on the membrane (or film) in both directions.

**Figure 2:**
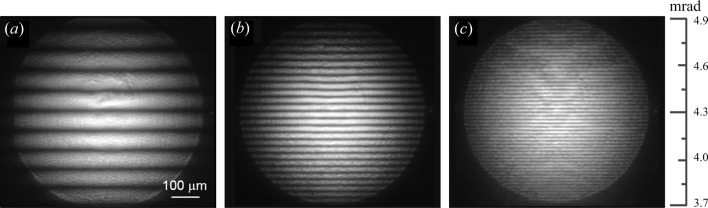
Reflecto-interferograms from Si_3_N_4_ membranes with thicknesses of (*a*) 200 nm, (*b*) 500 nm and (*c*) 1000 nm. The 100 µm scale corresponds to 224 µrad.

Fig. 2 ▸ presents interference patterns recorded using X-rays with an energy of 14.4 keV for membranes of thickness 200 nm, 500 nm and 1000 nm at an inclination angle θ equal to ∼4.3 mrad; the fringe spacing Δθ in the central part of the interferograms for these membranes is 174 µrad, 70 µrad and 35 µrad, respectively. From the experimental interferograms, it is clear that the thicker the membrane, the shorter the period of the fringes. The fringe period of all the membranes under study is in very good agreement with the calculations for expected thickness of membranes, which were performed using the equation (3).

**Figure 3:**
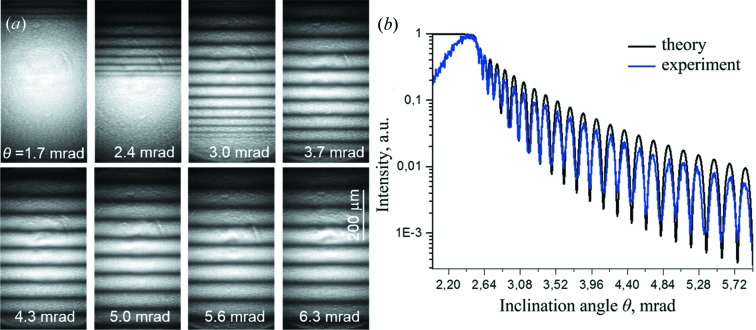
(*a*) Interference patterns registered for a 200 nm membrane at different inclination angles with 14.4 keV X-rays. The 200 µm scale corresponds to 448 µrad. (*b*) The experimental reflectivity curve obtained by combining interferograms recorded at membrane inclination angles from 2.4 mrad to 5.6 mrad.

Some notable interference patterns for a 200 nm-thick membrane observed at different inclination angles are shown in Fig. 3 ▸(*a*). As already shown above, the film thickness determines the distribution of the fringes, which can be calculated with equation (2). From this equation it follows that the period of the fringes varies with the grazing angle: the larger the angle, the larger the period asymptotically reaching a constant value. As can be seen in Fig. 3 ▸(*a*), fringes start to appear at angles above the critical angle θ_c_ = 2.6 mrad, and the distance between the fringes is not identical and it increases with increasing grazing angle. For example, for θ = 3 mrad, the fringe spacing Δθ is approximately 110 µrad and for θ = 5.6 mrad it is about 190 µrad. At angles θ > 4.3 mrad, the fringe pattern is becoming symmetrical with almost equal fringe spacing at the angular range produced by the CRL. The thickness of the membrane, obtained as a result of calculations according to equation (3), corresponds to the declared thickness of the membrane structure and it confirms the correct operation of the proposed reflecto-interferometer.

An experimentally measured reflectivity curve for 200 nm-thick Si_3_N_4_ membrane obtained by combining interferograms, which were recorded at the membrane inclination angles from 2.4 to 5.6 mrad, is presented in Fig. 3 ▸(*b*). This curve was formed using the following steps: a cross section of the intensity profile was extracted from each interference pattern, afterwards the intensity of the lens background was subtracted from the intensity of the received profiles and then they were stitched to obtain a complete reflectivity curve. It can be seen from Fig. 3 ▸(*b*) that the period of the experimental fringes corresponds to the period of the theoretical reflectivity curve calculated for the 200 nm-thick membrane. The difference in the contrast of the fringes in the experimental and theoretical reflection curves can be explained by the membrane roughness since in the theoretical model the interface is smooth.

### 3.2. Test of a gold strip on a 500 nm Si_3_N_4_ membrane with two-dimensional CRLs   

To demonstrate the spatial resolution of the proposed reflecto-interferometer, we experimentally investigated a 500 nm-thick membrane on which a gold strip was deposited. The gold strip was applied to the back side of the membrane by sputtering gold through an electron microscopy slot grid in the shape of a rectangle with a width of 250 µm and a length of 1.5 mm. According to deposition parameters, the thickness of the gold layer was around 8 nm. In addition, because of the deposition-process peculiarities, it cannot be excluded that the thickness of the gold layer toward to the edge of the strip is gradually decreasing.

For this test, we used 30 two-dimensional beryllium lenses with a parabola apex radius *R* of 50 µm, which focused 12 keVX-ray radiation to a focal spot of 0.31 µm × 6 µm at a distance *L*_1_ equal to 36 cm. The numerical aperture of the CRL was about 0.7 mrad; therefore to observe the reflective beam in full, the CCD camera was placed at a distance *L*_2_ = 64 cm from the sample. The membrane inclination angle θ was chosen to be 8.7 mrad. The footprint of the focused beam on the sample was below 100 µm along the beam and 6 µm horizontally, and the exposure time was 0.5 s. According to numerical calculations performed for this inclination angle, a focused beam reflected from a membrane with a gold layer of 8 nm underneath creates an interference pattern with interference fringes of minimal intensity. The deviation of the thickness of the gold layer from 8 nm leads to a significant increase in the intensity of interference fringes and to a decrease in their visibility. According to our estimates, the intensity of the interference fringes is sensitive to deviations in the layer thickness at a level of 1 nm.

**Figure 4:**
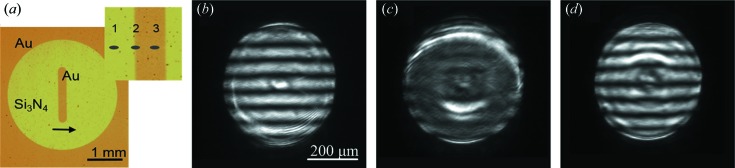
(*a*) Image in the optical microscope of the Au strip on the membrane. The inset shows locations where reflecto-interferograms were recorded crossing the Au edge. (*b*) Interferogram detected at position 1 on the membrane/air interface. (*c*) Interferogram recorded at position 2 at the boundary membrane/air and membrane/gold/air interface. (*d*) Interferogram registered at position 3 on the membrane/gold/air interface at the centre of the gold strip.

During this test the gold strip was scanned through a focused beam. The optical microscopy image of the membrane and the gold layer is depicted in Fig. 4 ▸(*a*). The arrow in this image shows the scanning direction of the sample; the scanning step was 50 µm. The Fig. 4 ▸(*a*) inset shows locations, indicated as 1, 2 and 3, where the most representative interferograms were recorded, which are presented in Figs. 4 ▸(*b*)–4 ▸(*d*). These positions correspond to reflections from the free-hanging membrane only, from the boundary membrane/air and membrane/gold/air interfaces, and from the middle of the gold strip.

The interference fringes are clearly visible in the images shown in Figs. 4 ▸(*b*) and 4 ▸(*d*). The angular fringe spacing is uniform and is about 98 µrad, which agrees with the calculated value, using equation (3) for a membrane with a thickness of 500 nm. It should be noted that the interference fringes in Figs. 4 ▸(*b*) and 4 ▸(*d*) are shifted from each other by almost half the period, while their intensities practically coincide. The result of equality of intensities confirms that the thickness of the Au layer is 8 nm, which was predicted during the deposition process and is also demonstrated by computer simulations. The reason for the shift of the fringes is the phase difference of the waves reflected from the interfaces between the membrane/air and the membrane/gold/air, which was confirmed by simulations as well.

The interference pattern recorded at the edge of the gold strip is shown in Fig. 4 ▸(*c*), from which it can be seen that the interference fringes are less visible or even almost completely disappeared. As mentioned above, the horizontal focus was 6 µm, so in position 2 in Fig. 4 ▸(*a*) both interfaces, membrane/air and membrane/gold/air, were equally illuminated. The interference pattern was destroyed because of the fact that the interference fringes from these interfaces are in antiphase and the corresponding interference patterns had almost the same intensities, therefore mixing these patterns leads to destructive interference. It should also be noted that in the centre of the interferograms in Figs. 4 ▸(*c*) and 4 ▸(*d*) a slight bending of the fringes can be observed, which is most likely to be because of the radiation damage of the gold layer, possibly because of its detachment from the membrane.

### 3.3. Test of a gold strip on a 1000 nm Si_3_N_4_ membrane with linear CRLs   

To reduce the effect of the radiation damage of the gold strip deposited on the underside of the membrane, which we noticed in the previous experiment, we tested the reflecto-interferometer employing a linear CRL. For use in this test, 36 linear (one-dimensional) beryllium lenses with a parabola apex radius *R* of 200 µm, a geometrical aperture of 1 mm and a length of 3 mm were stacked as a CRL. The linear CRL focuses X-rays in one direction but, since the source size is smaller in the vertical direction, the CRL was oriented in such a way as to also focus the X-rays in the vertical direction. At a photon energy of 12 keV, the CRL generated a convergent beam with a spot size of 0.9 µm at an imaging distance *L*_1_ that was equal to 1.2 m, which was where the sample was placed. The CRL numerical aperture at this energy was 0.6 mrad, so the sample was illuminated by a focused beam with angular range of the incident X-rays corresponding to the numerical aperture of the CRL.

In this test, we used a 1000 nm-thick membrane on which a gold strip was deposited by exactly the same procedure as described above. Therefore, a similar thickness and shape of the edges of the gold layer was expected. The CCD detector was placed at a distance of 85 cm from the sample. The footprint of the focused beam on the sample was 1.5 mm in the horizontal direction, which was limited by the slits, and less than 100 µm in the direction of the beam for all inclination angles of the membrane used in this test.

**Figure 5:**
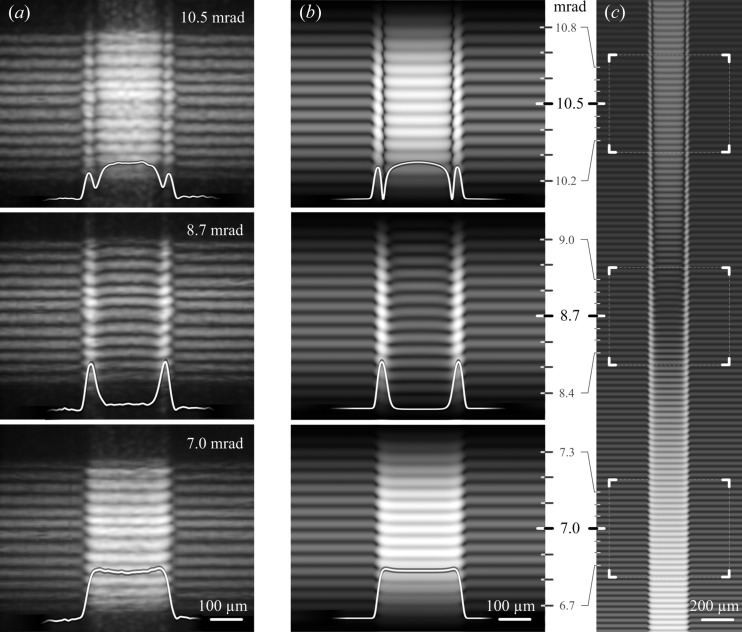
(*a*) Experimental reflecto-interferograms recorded at different angles of 7.0 mrad, 8.7 mrad and 10.5 mrad. (*b*) Numerically simulated reflecto-interferograms at the inclination angles corresponding to experimental data. (*c*) Calculated interferogram in a wider range of inclination angles.

During the experiment, a set of interferograms was obtained while changing the membrane inclination angle from 3.5 mrad to 12.2 mrad in steps of 0.87 mrad. The critical angles θ_c_ for gold and Si_3_N_4_ at the selected energy are about 6 mrad and 3 mrad, respectively. Some characteristic interference patterns recorded at inclination angles of 7.0 mrad, 8.7 mrad and 10.5 mrad are shown in Fig. 5 ▸(*a*) from bottom to top, respectively. Each of these experimental patterns demonstrates reflections from the membrane/air and membrane/gold/air interfaces simultaneously. The reflection from the gold strip is in the central part of the interferogram and reflections from the membrane are in the left and right parts. The implementation of linear CRLs eliminates the process of scanning the sample in the transverse direction, while maintaining a high spatial resolution. The period of interference fringes corresponds completely to a Si_3_N_4_ membrane with a thickness of 1000 nm, while the observed irregularity in the fringe width is associated with the CRLs aberrations and does not affect the accuracy of the thickness determination. The observed modulation of the average intensity over one period of fringes and their relative shifts in the vertical direction are determined by the reflection from the gold layer. In each interferogram, the sketch of the horizontal cross-section, showing the average intensity over one period of fringes, is drawn by a white line. For example, the increase in the intensity caused by the gold layer, as well as the shift of the interference fringes, are observed in the bottom image obtained at an inclination angle of 7 mrad. It should be noted that a slight bending of the interference fringes near the edges of the gold strip is determined by some variation in its thickness in these areas.

The influence of the variation in the thickness of the gold layer near the edges of the strip on the interference pattern is particularly noticeable in the middle image in Fig. 5 ▸(*a*), when the angle of the membrane inclination is 8.7 mrad. Apart from the bending of the fringes, a significant increase in the intensity at the edges of the gold strip is observed; whereas the interference fringes produced by the central part of the gold strip have a minimum average intensity, which is almost the same as that of the fringes collected from the membrane area. This result allows us to estimate that the thickness of the gold layer in the central part is equal to about 8 nm, which coincides with the thickness determined in the test using two-dimensional lenses. In addition, the characteristic bending of fringes and their high brightness at the gold-strip edges indicate a smooth decrease in the thickness of the layer.

A further increase in the membrane inclination angle again leads to an increase in the intensity of the interference fringes produced by the central part of the gold strip. As for fringes at the edges of the strip, where the gold layer is thinner, they have comparable intensity; however, two narrow dark areas appear near the edges. The presence of regions with such a low intensity of fringes at a given membrane inclination angle accurately indicates the corresponding layer thickness. For instance, this effect can be seen in the top image in Fig. 5 ▸(*a*), where the membrane inclination angle is 10.5 mrad. In this case, the fringes with lower intensity correspond to 6 nm thickness of the gold layer in the considered areas.

Numerically simulated reflecto-interferograms at inclination angles of 7.0 mrad, 8.7 mrad and 10.5 mrad are presented in Fig. 5 ▸(*b*). The images were calculated taking into account the CRL optical properties including the same numerical aperture as in the experiment. The model of the gold layer used in the simulation represents a thin strip, which has a Gaussian blur at the edges. Adjustment of the strip dimensions (thickness and width) and variation of the Gaussian filter parameters enable us to achieve the best agreement of the calculated and experimental interferograms. As a result, it not only allows us to determine very accurately the thickness of the gold layer but also allows us to reconstruct its cross-section profile. According to our simulations, the exact thickness of the gold layer in the middle of the strip was 8.3 nm ± 0.1 nm, while at edges of the strip it gradually decreased to zero. The width of the strip was about 250 µm (FWHM) which corresponds to the size of the electron-microscopy slot grid, which was used in the process of gold deposition. Fig. 5 ▸(*c*) shows a fragment of the interferogram calculated, taking into account a linear CRL which has a significant larger numerical aperture than the one used in the experiment. The areas indicated by square brackets correspond to the angles measured experimentally, where it is clearly seen that computer modelling is in good agreement with the experimental data. The extended range of angles provides a more informative representation of the conversions of interference fringes, which were observed experimentally with a discrete change in the membrane inclination angle.

### 3.4. Study of X-ray reflecto-interferometer temporal resolution on 870 nm-thick resist film   

**Figure 6:**
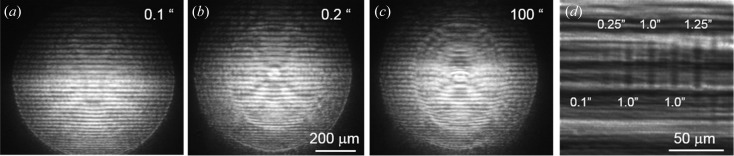
(*a*)–(*c*) Experimental reflecto-interferograms recorded at different exposures. (*d*) X-ray topogram of the damaged structure.

The positive photoresist S1811 was sputtered on a 540 µm-thick Si substrate. The estimated thickness of the resist layer was 870 nm in the centre of the Si wafer and 760 nm near the borders. To focus the 12 keV X-ray beam, 35 two-dimensional beryllium parabolic lenses with a parabola apex radius of 50 µm were used. The lens imaging distance *L*_2_ was 32 cm, which was where the sample was located. The CCD was placed at a distance of 45 cm downstream of the sample. It turns out that the resist is very sensitive to radiation damage. The sample inclination angle θ was chosen to be 2.63 mrad. An exposure of more than 0.1 s was sufficient to initiate the damage to the sample. The interference patterns recorded at different exposures are presented in Figs. 6 ▸(*a*)–6 ▸(*c*). The angular fringe spacing is about 58 µrad, which is in very good agreement with the film thickness in the centre of the wafer. The interference pattern in Fig. 6 ▸(*b*) begins to degrade, resulting in some deformation of the fringes. In Fig. 6 ▸(*c*) we show the interference image obtained after 100 s continuous exposure of the film. It can be clearly seen that the fringe spacing increases and the fringe contrast reduces, meaning that the resist-layer thickness, as well as its density, changed significantly after the X-ray illumination. This reflects the losses of the material caused by the initial out-gassing of polymers. The effect of degradation was reduced by employing an interferometer based on a combination of two-dimensional and linear lenses, which will be described in a future article.

Moving the lens out of the beam, we performed phase-contrast topography of the resist layer in the reflection mode. The X-ray topogram is shown in Fig. 6 ▸(*d*), where the radiation damage is clearly manifested at exposures of more than 0.1 s. It is also possible to see the beam dimensions with which the resist was exposed. It can be noted that the reflection is not uniform and has a striking contrast, which indicates the uneven thickness of the resist.

## 4. Discussion and conclusion   

In the present research, we demonstrated a new intensity-division X-ray reflecto-interferometer based on refractive optics. The idea of the proposed interferometer is to use a focused X-ray beam produced by the CRL, which is reflected from parallel flat surfaces of the sample thereby creating an interference pattern over a wide angular range. In fact, the suggested concept of the reflecto-interferometry utilizes the same optical principles of reflection as in classical X-ray reflectometry techniques. Moreover, it should be pointed out that it is rather complementary to the conventional X-ray reflectometry. Nevertheless, the suggested interferometer using the imaging approach has evident advantages over classical X-ray reflectivity.

The first obvious advantage of the implemented setup is the possiblity to measure the interference pattern, which is in fact specular X-ray reflectivity, in a very simplified experimental setup without the need to rotate the specimen or detector. Thus, the X-ray reflecto-interferometer does not require mechanics with a high angular resolution. The functional capabilities of the interferometer were tested by studying Si_3_N_4_ membranes of different thicknesses in the range from 200 nm to 1000 nm. The experimentally obtained reflecto-interferograms are in good agreement with those calculated, and the distance between fringes in the interference patterns correspond to the thickness of the tested membranes.

The recording of the interference pattern in a single shot allows a fast analysis of the films, which is especially important for radiation-sensitive materials, including organic and biological films. The temporal resolution of the proposed approach is limited only by the exposure time, which is necessary to obtain an image of the interference fringes on the detector. Typically, the temporal resolution is a few milliseconds, depending on the X-ray flux and the sensitivity of the detector. Practically applied, this method can be used as an *in situ* film growth study, providing real-time information on the film thickness, density and roughness during the deposition process. It is worth noting that such an *in situ* study is impossible in the traditional reflectivity approach, where the time resolution is strongly limited by the data-acquisition method itself and usually ranges from a few minutes to several hours. The temporal resolution of the interferometer was demonstrated by studying the 870 nm resist layer on the Si substrate. In our experiments, we observed a deterioration of the interference pattern associated with the radiation damage of the resist layer within 10 ms of exposure at the incident flux 10^13^ photons s^−1^.

The second feature of the proposed reflecto-interferometer is the spatial resolution, which is achieved because of focusing by CRLs, allowing one to probe different regions on the film with an accuracy of the order of several tens of micrometres or even less. This property is very important for studying samples with high roughness and extremely small lateral size, and is also necessary for the local analysis of the film. Moreover, the probing area can be quickly changed without modifying the experimental setup by moving the sample out of the focal spot of the lens causing defocusing. The high spatial resolution of the interferometer was demonstrated on interferograms recorded from an 8 nm-thick gold strip deposited on an Si_3_N_4_ membrane. It was shown that the interference pattern is very sensitive to deviations in the thickness of the layer, resulting in not only the ability to determine very accurately the average thickness of the gold layer but also the ability to reconstruct its cross-section profile. The obtained results enable one to consider the reflecto-interferometer as a sensitive tool for studying non-uniform and heterogeneous samples as well as complex structured thin films and multilayer systems.

Let us estimate the minimum and maximum thickness of films, which can be investigated by means of the lens-based reflecto-interferometer, which records the interference pattern in a single shot. Regarding the minimum thickness of film, as mentioned earlier, it is determined from the ratio of the CRL numerical aperture to the angular distance between two successive interference fringes but only when this ratio is equal to one. Considering 100 beryllium lenses with a parabola apex radius of 50 µm at the energy 12.1 keV, we can satisfy the above criterion, while maintaining a good balance between the lens imaging distance and the length of the lens. This means that it is possible to study films with a thickness of 40 nm. Using 180 polymethyl methacrylate (PMMA) lenses with a parabola radius of 5 µm at an energy of 20.5 keV, 18 nm-thick films can be studied. The proposed scheme of the reflecto-interferometer also allows us to investigate even thinner films using a combination of several interference patterns obtained by successive scanning.

If we refer to maximum thickness of the film, which can be analysed with our reflecto-interferometer, it is limited by the instrumental angular resolution, defined as the ratio of the spatial resolution of the detector to the distance from the film to the detector. For example, considering a detector with a spatial resolution of 1 µm, which is located 1 m from the film, it is possible to achieve a typical instrumental angular resolution of about 1 mrad, which means that the reflecto-interferometer put forward in this article can be used to study films with a thickness of several tens of micrometres. It is known that the study of thin films is well developed in classical X-ray reflectometry, while analysis of films with a thickness of more than 1 µm is a challenging task. Certainly it makes our technique more attractive; however, it should be noted that, despite the excellent instrumental capabilities, the study of thick films is a difficult task. The point is that when considering thick films, it is necessary to take into account the absorption of X-rays in the film material, which leads to a significant deterioration in the quality of the formed interference fringes. Therefore, in practice, for most films, the maximum thickness does not exceed a few micrometres.

The new reflecto-interferometry method opens up a wide horizon for the analysis of thin-film and multilayer systems. Because of the focusing properties of compound refractive lenses, completely new research prospects are emerging both in the field of condensed-matter physics and in the field of materials science and biological applications, and also for carrying out industrial research with a fundamentally new spatial and temporal resolution.
